# The Impact of Epidemic Violence on the Prevalence of Psychiatric Disorders in Sao Paulo and Rio de Janeiro, Brazil

**DOI:** 10.1371/journal.pone.0063545

**Published:** 2013-05-08

**Authors:** Wagner Silva Ribeiro, Jair de Jesus Mari, Maria Inês Quintana, Michael E. Dewey, Sara Evans-Lacko, Liliane Maria Pereira Vilete, Ivan Figueira, Rodrigo Affonseca Bressan, Marcelo Feijó de Mello, Martin Prince, Cleusa P. Ferri, Evandro Silva Freire Coutinho, Sérgio Baxter Andreoli

**Affiliations:** 1 Universidade Federal de São Paulo, Departmento de Psiquiatria, Sao Paulo, Brazil; 2 King’s College London, Health Service and Population Research Department, Institute of Psychiatry, London, United Kingdom; 3 Fundação Oswaldo Cruz, Escola Nacional de Saúde Pública, Rio de Janeiro, Brazil; 4 Universidade Federal do Rio de Janeiro, Instituto de Psiquiatria, Rio de Janeiro, Brazil; Max Planck Institute of Psychiatry, Germany

## Abstract

**Background:**

Violence and other traumatic events, as well as psychiatric disorders are frequent in developing countries, but there are few population studies to show the actual impact of traumatic events in the psychiatric morbidity in low and middle-income countries (LMIC).

**Aims:**

To study the relationship between traumatic events and prevalence of mental disorders in São Paulo and Rio de Janeiro, Brazil.

**Methods:**

Cross-sectional survey carried out in 2007–2008 with a probabilistic representative sample of 15- to 75-year-old residents in Sao Paulo and Rio de Janeiro, Brazil, using the Composite International Diagnostic Interview.

**Results:**

The sample comprised 3744 interviews. Nearly 90% of participants faced lifetime traumatic events. Lifetime prevalence of any disorders was 44% in Sao Paulo and 42.1% in Rio de Janeiro. One-year estimates were 32.5% and 31.2%. One-year prevalence of traumatic events was higher in Rio de Janeiro than Sao Paulo (35.1 vs. 21.7; p<0.001). Participants from Rio de Janeiro were less likely to have alcohol dependence (OR = 0.55; p = 0.027), depression (OR = 0.6; p = 0.006) generalized anxiety (OR = 0.59; p = 0.021) and post-traumatic stress disorder (OR = 0.62; p = 0.027). Traumatic events correlated with all diagnoses – e.g. assaultive violence with alcohol dependence (OR = 5.7; p<0.001) and with depression (OR = 1.7; p = 0.001).

**Conclusion:**

Our findings show that psychiatric disorders and traumatic events, especially violence, are extremely common in Sao Paulo and Rio de Janeiro, supporting the idea that neuropsychiatric disorders and external causes have become a major public health priority, as they are amongst the leading causes of burden of disease in low and middle-income countries. The comparison between the two cities regarding patterns of violence and psychiatric morbidity suggests that environmental factors may buffer the negative impacts of traumatic events. Identifying such factors might guide the implementation of interventions to improve mental health and quality of life in LMIC urban centers.

## Introduction

There is a strong relationship between economic development and better health profiles of populations across different countries. The undergoing economic and social development in low- and middle-income countries (LMIC) has promoted changes in the patterns of health and disease, the so called epidemiological transition [1], resulting in a significant reduction in general mortality, as well as infant mortality, an increase in life expectancy, and the displacement of infectious and communicable diseases as primary causes of morbidity and mortality. Non-communicable degenerative and/or chronic diseases, such as mental disorders, as well as external causes, such as violence and injuries, are now becoming more prominent in LMIC.

In Brazil, important demographic shifts have occurred in the second half of the twentieth century. The country’s population increased from 52 million in 1950 to 170 million in 2000. During the same period, Brazil changed from a predominantly rural to a predominantly urban society, with the proportion of people living in urban settings increasing from 36% in 1950 to 81% in the year 2000 [2], [3]. As a result of social and economic developments, Brazil’s epidemiological profile has changed, especially in the most urbanized regions. In 2007, non-communicable diseases accounted for 72% of all deaths in the country, whereas 10% of deaths were attributed to infections diseases [4]. Neuropsychiatric diseases alone accounted for 34% of years lost due to disability (YLD) and 19.6% of disability-adjusted life years (DALY) [5].

Even though life conditions have improved over the last decades due to the economical growth and to governmental interventions, Brazil is still one of the most unequal countries in the world. Regarded as one of the ten largest economies [6], the country is, nonetheless, 73^rd^ in the human development ranking [7] and its Gini-coefficient of inequality, which measures economical disparities, is the 10^th^ highest in the world [8]. Alongside changes in wealth and social disparities, levels of violence and other external causes have also increased substantially [9]. In Brazilian urban centers, homicide rates, which are usually taken as a proxy measure for general levels of violence, increased continuously through 2003, upon which they began to decline, mostly as a result of a new legislation that restricted access to firearms in the country [10]. Nonetheless, homicides were responsible for 36% of all deaths from external causes in 2007 [11]. Notwithstanding these figures, estimates on the prevalence of non-fatal violence are lacking.

Population-based surveys carried out in Brazil have shown that psychiatric disorders are highly prevalent in the country. A study conducted in three metropolitan areas in the late 1990s found the lifetime prevalence of any psychiatric disorder to vary from 20% to 50% [12]. Another study carried out in Sao Paulo in the early 2000s found that 46% of respondents had a lifetime mental disorder, and that nearly 27% had a one-year psychiatric disorder [13]. None of these studies explored the possible relationship between violence and mental health. A Brazilian population survey which has been published recently [14] also found significant levels of mental disorders among residents of a major metropolitan area in Brazil (30% one-year prevalence). This study explored the association of mental disorders with some crime-related traumatic events that are thought to be common in Brazil, and concluded that such events correlated with an increased prevalence of all classes of disorders. Even though the authors suggest that these results may illustrate what happens in other metropolitan areas in terms of prevalence and patterns of mental health morbidity, they recognize that it may be difficult to generalize their findings to other areas in Brazil and elsewhere. Moreover, the study did not investigate the full spectrum of traumatic events commonly presented in Brazilian urban centers, such as witnessing shoot-outs or being a victim of stray bullets and traffic accidents, among others, which might increase the likelihood of developing mental disorders. Previous studies showed an association between traumatic events and mental health problems in low and middle-income countries for different populations [15], [16], [17], [18], [19]. Most of these studies were designed to assess specific traumatic events in specific populations, such as intimate partner violence among women, domestic violence among children, or traumatic events in conflict/post-conflict settings. The few studies that were carried out in the general population were designed to assess the prevalence and correlates of post-traumatic stress disorder, and did not examine the association of traumatic events with other common mental disorders [20].

A comparison between Sao Paulo and Rio de Janeiro, which are the two largest urban centers in Brazil, shows that both cities have faced a rapid and disorganized urbanization process through the past decades, which led to a dramatic social and economic inequality. Despite their similarities, available data suggests that frequency and patterns of exposure to violence have differed substantially between the two cities: in 2007, for instance, homicide rates in Rio de Janeiro were almost three times as high as in Sao Paulo [21]. The comparison between these two cities can shed light on the relationship between traumatic events and psychiatric morbidity, and the role of mediating geographical and social factors, as it allows for the comparison of prevalence estimates in areas with different levels of exposure to traumatic events.

The aim of this survey was to study the relationship of exposure to violence and other traumatic events with the prevalence of psychiatric disorders in the two largest urban centers in Brazil: Sao Paulo and Rio de Janeiro. The two sites were chosen because of their differences in frequency and patterns of exposure to traumatic events, especially to violence, alongside with their similarities regarding urbanization processes and socio-economic inequality. Given the similarities and differences between the two cities, we hypothesized that even though exposure to traumatic events would be prevalent in the two sites, those living in Rio de Janeiro would report a much higher exposure to violent events. We also hypothesized that, due to the higher exposure to violence, prevalence of psychiatric disorders would be significantly higher in Rio de Janeiro than in Sao Paulo.

## Methods

### Ethical Statement

The Ethics Committee of the Federal University of Sao Paulo analyzed and approved the study’s protocol, including informed consents, questionnaire, procedures for recruitment and interview of participants, as well as mechanisms for protecting participants’ privacy, integrity and rights. Respondents were interviewed only after they signed informed consents. When the participants were 15 to 17 years old, their parents or legal substitutes also signed the informed consents in order to authorize them to be interviewed. Subjects who matched diagnostic criteria were offered a referral to outpatient clinics at the Federal University of Sao Paulo and Federal University of Rio de Janeiro.

### Setting

Sao Paulo comprises 11 million inhabitants, being the largest city in Brazil. It is the most important industrial, commercial and financial center in the country. In 2007, its gross domestic product (GDP) was estimated to be around 180 billion US dollars, and the GDP per capita was nearly 16 thousand US dollars/year. With 6 million inhabitants, Rio de Janeiro is the second largest city in the country. Its economy is predominantly based on services and tourism. The GDP was estimated to be 70 billion US dollars in 2007, and the GDP per capita was around 13 thousand US dollars. The two cities present high levels of socio-economic inequality and violence. The average homicide rate in 2007 was 15.0/100,000 inhabitants in Sao Paulo and 40.1/100,000 in Rio de Janeiro [21]. In the same year, the average homicide rate in the country was 25.2/100,000 inhabitants [21].

### Design and Sampling Procedures

We carried out a one-phase cross-sectional survey with a representative sample of the population aged 15 to 75 years in Sao Paulo and Rio de Janeiro. In order to increase the likelihood of identifying victims of violence, the cities were stratified according to their homicide rates, as follows: firstly, the administrative areas in each city were ranked according to their homicide rates. In each city, an administrative area is a geographic unity that comprises a number of neighborhoods, which usually have similar characteristics, within a specific region. There are 96 administrative areas in Sao Paulo, and 33 in Rio de Janeiro. The administrative areas were then grouped into six strata (1 =  less than 10 homicides/100,000 inhabitants; 2 = 10.01 to 20; 3 = 20.01 to 30; 4 = 30.01 to 40; 5 = 40.01 to 50; 5 = 50.01 to 60; and 6 = more than 60 homicides/100,000 inhabitants). In the second stage, all the census sectors within each stratum were mapped. A number of census sectors were randomly selected within each stratum. The number of census sectors varied from 4 to 18 according to the population size within each stratum. In the third stage, 43 households (Sao Paulo) or 30 households (Rio de Janeiro) were randomly selected within each census sector on the base of odd random numbers. In each selected household all dwellers aged 15 to 75 years were enumerated, and one of them was randomly selected based on the Kish's method [22].

### Sample Size

Precision calculations indicated that a sample size of around 850 interviews would allow estimation of lifetime prevalence of post-traumatic stress disorder of 10%, within a 95% confidence interval. Due to an expected refusal rate of 20%, and in order to increase the likelihood of identifying post-traumatic stress disorder cases, the three strata with the highest homicide rates were oversampled, resulting in 1500 interviews. In Sao Paulo, we expected to identify approximately 120 current cases to be referred to a case-control study [23] and to a clinical trial [24] on post-traumatic stress disorder (PTSD). As we expected a 5% one-year prevalence of PTSD, we decided to increase the sample size to 3,000 interviews to allow for the identification of the required PTSD cases.

### Measures

Psychiatric disorders were assessed via the Composite International Diagnostic Interview version 2.1 (CIDI 2.1). The CIDI 2.1 is a standardized and fully structured interview that provides psychiatric diagnoses according to the International Classification of Diseases, 10^th^ edition (ICD-10), and the Diagnostic and Statistical Manual of the American Psychiatric Association, 4^th^ edition (DSM-IV) [25], [26]. In this article, it was decided to present only the DSM-IV diagnoses provided by the questionnaire.

A Portuguese version of the CIDI 2.1 has been previously validated and adapted to the Brazilian social and cultural context [27]. The following sections of the questionnaire were applied: alcohol abuse and dependence; depressive disorders; panic disorder; phobic disorders (social, specific and agoraphobia); generalized anxiety disorder, obsessive-compulsive disorder; and post-traumatic stress disorder.

Exposure to violence and other potentially traumatic events were assessed through a list of traumatic events adapted from the post-traumatic stress disorder section of the CIDI 2.1. Twenty new events were added to the 11 items of the original list so as to cover most of the traumatic events experienced by individuals in Brazilian urban centers. The episodes were then scored on the frequency, intensity, and the first and the last time of occurrence. Table 1 shows the original list of traumatic events from CIDI 2.1 and the 20 new events added in this study.

**Table 1:** Original list of traumatic events in CIDI 2.1 and updated list with additional traumatic events.

Traumatic events form CIDI 2.1	Traumatic events added in the study
1. War experience	1. Being attacked without a weapon
2. Life-threatening accident	2. Being attacked with a weapon*
3. Fire, flood of other natural disaster	3. Being kidnaped or held captive*
4. Witnessing someone being badly injured of killed	4. Fast kidnap†
5. Rape	5. Death threats
6. Sexual molestation	6. Being victim of conflict between gangs/drug dealers
7. Being physically attacked or assaulted	7. Being beaten-up by parents/relatives
8. Being threatened with a weapon, held captive or kidnapped*	8. Being beaten-up by an intimate partner
9. Being tortured of victim of terrorism	9. Being beaten-up by anyone else
10. Other extremely stressful or upsetting event	10. Having the house broken into while at home
11. Events of the list happening with a close person	11. Having the house broken into while not at home
	12. Blackmailing telephone calls‡
	13. Car/motorcycle accidents
	14. Witnessing a bank robbery
	15. Witnessing a shoot-out or being victim of stray bullet
	16. Witnessing domestic violence in childhood
	17. Seeing or touching a corpse unexpectedly
	18. Witnessing atrocities, slaughter, massacre
	19. Human-made disaster
	20. Direct consequences of crime organization’s attacks¥
	21. Indirect consequences of crime organization’s attacks¥

Notes:

* Event 8 from the original list was disaggregated into two separate events (2 and 3 in the list of added events).

† In the fast kidnap, the person is held captive for several hours and taken to withdraw cash from ATMs.

‡ In this event, criminals call someone and pretend they kidnaped and will kill one of his/her relatives if he/she does not transfer them a certain amount of cash.

¥ In 2006, criminal organizations in Sao Paulo and Rio de Janeiro perpetrated a series of random attacks, burning buses and buildings, as well as murdering policemen and other low-enforcement personal. The population was affected directly (by being present in the situation) and/or indirectly (by feeling upset due to the terror that spread throughout the cities during the nearly one-month period the attacks happened.

### Procedures

Face-to-face interviews were carried out by a team of lay interviewers under the auspices of the Brazilian Institute of Public Opinion and Statistics (www.ibope.com.br), one of the largest Brazilian independent population research institutes. The interviewers were trained and supervised by the study investigators. In order to optimize response rates, interviewers made up to ten visits to the selected households.

### Statistical Analysis

Data were weighted to account for oversampling of people living in the most violent strata and for differential probability of selection, in order to allow for the estimation of population-level prevalence estimates. Weighted prevalence estimates of traumatic events and psychiatric outcomes are presented. Standardized prevalence estimates of psychiatric disorders were also estimated in order to allow for comparisons between the two cities. The standardization was based on the current gender and age distribution of Sao Paulo’s population (http://www.seade.sp.gov.br). To take account of the study design, the odds ratio and 95% confidence intervals were estimated using STATA’s survey data analysis commands, which have been developed for analyzing complex sample surveys and weighted data.

Analyses of frequency were performed to describe the sample characteristics. Prevalence estimates of traumatic events and of psychiatric disorders was estimated based on the proportion of interviewees who endorsed those events and matched diagnostic criteria. Bivariate associations between prevalence estimates and sample characteristics (e.g. city, gender, etc.) were assessed through 2×2 contingency tables (cross tabulations) and Chi-Square Tests of Associations.

Multivariate logistic regression analyses were performed to assess correlation of psychiatric disorders with demographic characteristics and number of traumatic events reported by participants. For each psychiatric disorder we ran a separate multivariate logistic regression analysis. We chose the one-year prevalence estimates as dependent variables in order to establish, as much as possible, a temporal relation between the outcomes and correlates. The following demographic characteristics were entered in these models as independent variables: setting (Sao Paulo vs. Rio de Janeiro); gender (male vs. female); age, in four categories (15–29 years; 30–44; 45–59; 60–75); education, as continuous variable (number of years in school); marital status (single, married, divorced, widowed); employment status, meaning being currently employed (no vs. yes); and migration status (no vs. yes). Number of traumatic events was included in this multivariate logistic regression analysis as it was expected to vary between cities and gender.

We also assessed the association between different types of traumatic events and psychiatric diagnoses. In order to establish a temporal relationship between exposure and psychiatric disorders, and to avoid reverse causality, only traumatic events that happened more than one year prior to the survey were included in the analyses as independent variables, whereas one-year estimates of psychiatric disorders were chosen as dependent variables. We created three binary variables (no vs. yes) that grouped traumatic events into three major types of events: assaultive violence, which includes direct personal trauma that involves interpersonal violence; other injury or shocking events, which includes other types of direct personal traumatic experiences; and sudden death or life-threatening injury of a close person. The literature has described direct exposure to traumatic experiences, especially those involving interpersonal violence, as risk factors for mental disorders, and also has shown that sudden death/life-threatening injury of close persons is highly prevalent [14], [17], [28].

We assessed the bivariate association between each type of traumatic events and each psychiatric disorder through 2×2 contingency tables (cross tabulations) and Chi-Square Tests of Associations. We also estimated the Odds Ratios of these associations through bivariate logistic regression analyses. After assessing the bivariate associations, we ran two multivariate logistic regression models for each type of traumatic events, as follow: Model 1 estimated the Odds Ratios of the association between types of traumatic events and psychiatric diagnoses, controlling for demographic characteristics (city, gender, age, marital status, education, employment status and migration history); Model 2 included the same demographic characteristics, plus the other two types of traumatic events, in order to adjust the Odds Ratios controlling for the types of traumatic events. Only the associations between types of traumatic events and psychiatric diagnoses that were statistically significant according to the bivariate analyses were included in the multivariate logistic regression models.

For one-year post-traumatic stress disorder we ran the bivariate and multivariate logistic regression analyses only in the subsample of respondents who reported at least one lifetime traumatic event, as exposure to traumatic events is mandatory to generate this diagnosis. For all statistical tests through this article we adopted a 5% significance level (p<0.05).

## Results

### Sample Characteristics

The final sample comprised 3744 interviews (2536 in Sao Paulo and 1208 in Rio de Janeiro), corresponding to response rates of 84.5% and 80.5%, respectively. The sample included more women than men in both cities (Table 2). Participants were slightly younger in Sao Paulo (mean = 39.5 years; SD = 15.1) as compared to Rio de Janeiro (mean = 42.4 years; SD = 16.1) (p<0.001). Most interviewees in both cities were married or cohabiting. Overall, education level was lower in Sao Paulo (mean = 8.3 years of education; SD = 4.2) than in Rio de Janeiro (mean = 9.8 years; SD = 4.4). Most of the participants were employed, and the proportion of migrants was much higher in Sao Paulo as compared to Rio de Janeiro (50% *vs.* 32%, p<0.001).

**Table 2:** Demographic Characteristics of Final Sample, Sao Paulo and Rio de Janeiro, Brazil, 2007–2008.

	Sao Paulo	Rio de Janeiro	
	N (%*)	N (%*)	P value†
**Gender**			
Male	1096 (41.9)	524 (43.4)	0.464
Female	1440 (58.1)	684 (56.6)	
**Age (years)**			
15–29	851 (31.3)	321 (26.3)	<0.001
30–44	873 (32.3)	633 (51.9)	
45–59	545 (24.3)	320 (26.6)	
60–75	267 (12.0)	202 (17.8)	
Mean (SD)	39.5 (15.1)	42.4 (16.1)	<0.001
**Marital status**			
Single	717 (28.3)	375 (31.4)	0.089
Married/cohabiting	1467 (56.8)	633 (51.9)	
Separated/divorced	228 (9.0)	129 (10.7)	
Widowed	124 (5.8)	71 (6.0)	
**Education (years of school)** ‡			
0–4	588 (20.0)	173 (14.6)	<0.001
5–8	706 (25.5)	284 (23.1)	
9–12	954 (38.9)	505 (41.1)	
13 or more	287 (15.6)	246 (21.2)	
Mean (SD)	8.3 (4.2)	9.8 (4.4)	<0.001
* Missing values*	*1*		
**Occupational status**			
Currently unemployed	977 (39.7)	509 (44.6)	
Currently employed	1559 (60.3)	699 (56.4)	0.055
**Migration history**			
Being born in the study site	1150 (49.9)	831 (31.3)	
Being a migrant	1385 (50.1)	377 (31.7)	<0.001
* Missing values*	*1*		

* Estimates presented are weighted.

† p-value based on Chi-Square test.

‡ Education = number of completed school years.

### Exposure to Violence and Other Traumatic Events

Lifetime exposure to any traumatic events was similar in the two cities (Table 3). Nonetheless, lifetime exposure to assaultive violence was higher in Rio de Janeiro (63.6%) than Sao Paulo (59.4%). In the assaultive violence category, physical assault with a weapon, kidnap, being victim of conflict between gangs/drug dealers, and sexual violence were more prevalent in Rio de Janeiro, whereas fast kidnap, where the person is held for a few hours to withdraw money from cash machines, was more prevalent in Sao Paulo. As compared to those living in Sao Paulo, respondents from Rio de Janeiro reported more natural and human-made disasters, witnessing a shoot-out or being a victim of stray bullet, seeing or touching a corpse, and witnessing atrocities, slaughter or massacres. On the other hand, respondents from Sao Paulo experienced more housebreaking when they were not at home.

**Table 3:** Exposure to Traumatic Events in Sao Paulo (SP) and Rio de Janeiro (RJ), 2007–2008*.

	Lifetime prevalence				12-month prevalence			
Traumatic events	SP	RJ			SP	RJ		
	% (95%CI)	% (95%CI)		P value	% (95%CI)	% (95%CI)		P value
***Assaultive violence***	***59.4 (57.0–61.8)***	***63.8 (60.8–66.90***		***0.024***	***9.5 (8.0–11.0)***	***11.4 (9.5–13.3)***		***0.115***
War experience	0.5 (0.01–8.9)	1.1 (0.5–1.7)		0.104	0	0.08 (0.0–2.4)		0.114
Being attacked without weapon	21.8 (19.7–23.8)	24.6 (22.0–27.3)		0.093	1.0 (0.5–1.5)	2.5 (1.5–3.4)		0.005
Being attacked with weapon	28.8 (26.6–31.0)	33.1 (30.2–36.1)		0.020	2.5 (1.7–3.2)	2.4 (1.5–3.3)		0.946
Being kidnapped, or held captive	0.7 (0.4–1.1)	1.5 (0.8–2.3)		0.048	0.1 (0.0–0.3)	0.08 (0.0–0.2)		0.840
Fast kidnap†	2.0 (1.2–2.8)	0.7 (0.2–1.2)		0.010	0.02 (0.0–0.6)	0		0.528
Torture/terrorism	0.8 (0.3–1.2)	2.2 (1.3–3.1)		0.003	0.1 (0.0–0.3)	0.2 (0.0–0.4)		0.814
Death threats	12.1 (10.5–13.7)	11.9 (9.8–13.9)		0.857	2.3 (1.5–3.1)	2.3 (1.4–3.2)		0.939
Conflict between gangs/drug dealers‡	1.2 (0.7–1.8)	3.0 (1.9–4.0)		0.002	0.3 (0.0–0.7)	0.6 (0.1–1.1)		0.324
Rape	1.3 (0.8–1.8)	2.3 (1.4–3.3)		0.044	0	0.008 (0.0–0.2)		0.114
Sexual molestation	2.5 (1.8–3.3)	4.9 (3.5–6.2)		0.001	0.004 (0.0–0.1)	0.02 (0.0–0.4)		0.219
Being beaten-up by parents/relatives	7.3 (6.1–8.6)	9.1 (7.2–10.8)		0.107	0.06 (0.2–1.0)	0.4 (0.1–0.7)		0.459
Being beaten-up by an intimate partner	6.8 (5.6–8.0)	6.8 (5.3–8.4)		0.992	0.9 (0.5–1.4)	0.9 (0.4–1.4)		0.885
Being beaten-up by anyone else than family/partner	3.7 (2.8–4.6)	4.2 (3.0–5.3)		0.523	0.2 (0.0 −1–0.4)	0.3 (0.02–0.6)		0.595
Having one’s house broken into while at home	9.0 (7.6–10.5)	7.3 (5.7–8.9)		0.128	1.2 (0.7–1.8)	0.8 (0.2–1.3)		0.242
Blackmailing telephone calls	8.7 (7.2–10.1)	12.0 (10.0–14.0)		0.006	3.0 (2.1–3.8)	3.4 (2.3–4.4)		0.584
***Other injury or shocking events***	***72.7 (70/7–75.0)***	***79.0 (75.9–81.9)***		<***0.001***	***12.8 (11.1–14.4)***	***23.7 (21.0–26.4)***		***<0.001***
Car/motorcycle accident	18.1 (16.2–20.0)	17.4 (15.0–19.7)		0.645	1.3 (0.7–1.8)	1.5 (0.7–2.3)		0.625
Accidents other than car/motorcycle	5.3 (4.2–6.4)	7.1 (5.4–8.7)		0.070	0.4 (0.1–0.8)	0.5 (0.04–1.0)		0.836
Fire, flood, natural disaster	7.5 (6.2–8.9)	10.3 (8.4–12.3)		0.016	0.9 (0.3–1.4)	0.6 (0.1–1.0)		0.375
Witnessing someone being killing or injured	27.1 (24.9–29.3)	28.2 (25.4–31.1)		0.524	3.4 (2.5–4.3)	4.4 (3.1–5.7)		0.222
Witnessing bank robbery	7.0 (5.6–8.3)	7.1 (5.5–8.8)		0.875	0.8 (0.3–1.3)	0.8 (0.2–1.4)		0.973
Witnessing a shoot-out or being victim of stray bullet	16.1 (14.3–17.9)	29.4 (26.5–32.2)		<0.001	2.0 (1,3–2.7)	11.7 (9.6–13.8)		<0.001
Witnessing domestic violence during childhood¥	16.2 (14.5–18.0)	17.5 (15.1–19.8)		0.404	0.3 (0.0–0.6)	0.0		0.223
Having one’s house broken into while not at home	14.2 (12.4–15.9)	10.4 (8.5–12.3)		0.006	1.2 (0.6–1.7)	1.5 (0.7–2.3)		0.486
Seeing or touching a corpse	31.0 (28.7–33.3)	37.5 (34.4–40.5)		0.001	4.6 (3.6–5.6)	10.3 (8.4–12.3)		<0.001
Witnessing atrocities, slaughter, massacre	7.7 (6.4–9.0)	11.5 (9.6–13.4)		0.001	1.0 (0.5–1.5)	2.2 (1.3–3.1)		0.014
Human-made disaster	2.2 (1.5–3.0)	4.3 (3.0–5.6)		0.004	0.4 (0.02–0.7)	0.4 (0.0–0.9)		0.782
Witnessing crime organizations’ attacksΔ	25.2 (23.1–27.4)	26.7 (23.9–29.5)		0.417	0.4 (0.2–0.5)	1.1 (0.4–1.7)		0.003
***Sudden death/life-threatening illness of a close person***	***47.1 (44.6–50.0)***	***49.7 (46.6–52.9)***		***0.194***	***4.7 (3.7–5.7)***	***7.4 (5.7–9.0)***		0.004
Sudden unexpected death of a close person	42.2 (39.8–44.6)	45.1 (42.0–48.2)		0.150	3.8 (2.8–4.7)	6.8 (5.2–8.2)		<0.001
Child with life-threatening illness or injury	9.6 (8.2–11.0)	9.1 (7.3–11.0)		0.702	1.0 (0.5–1.5)	0.8 (0.2–1.3)		0.579
***Any traumatic event***	***86.0 (84.4–87.7)***	***88.7 (86.7–90.6)***		***0.055***	***21.7 (19.6–23.7)***	***35.1 (32.1–38.1)***		***<0.001***

* Prevalence estimates presented are weighted estimates.

† In the fast kidnap the person is kidnaped and held captive for several hours to withdraw cash form ATMs.

‡ Conflicts between gangs/drug dealers refer to fights between rival groups, usually to control drug traffic areas in the slams.

¥ This includes events that occurred up to 12 years of age.

Δ In 2006 and 2007, crime organizations perpetrated a series of random gunshots, depredations and bus-burnings in the two cities.

**Figure 1:**
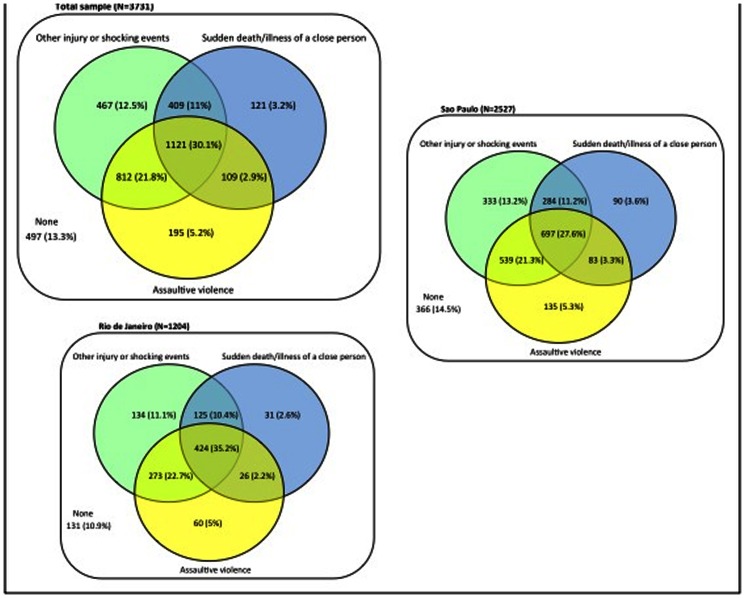
Figure 1 shows the patterns of exposure to three types of traumatic events (assaultive violence, other injury or shocking events, and sudden death/life-threatening illness of a close person) in Sao Paulo and Rio de Janeiro, Brazil. The diagram shows that, from the 3234 participants in the total sample who experienced at least one lifetime traumatic event, 1330 (41%) reported two types of traumatic events –906 (41.7%) in Sao Paulo and 427 (39.5%) in Rio de Janeiro, and 1121 (34.7%) experienced the three types of traumatic events –697 (32.1%) in Sao Paulo and 424 (39.5%) in Rio de Janeiro.

Most participants in this sample reported experiencing two or three types of traumatic events in their lifespan. Among those exposed, only a quarter reported just one type of event (26% in Sao Paulo and 20% in Rio de Janeiro). One-third of participants (32% in Sao Paulo and 40% in Rio de Janeiro) experienced the three classes of traumatic events. The Venn diagram in Figure 1 presents the patterns of exposure according to types of traumatic events.

One-year prevalence estimates of any traumatic events were higher in Rio de Janeiro (35.1%) than Sao Paulo (21.7%). One-year prevalence estimates of exposure to assaultive violence was similar in the two cities, with the exception of being attacked without a weapon, which was more prevalent in Rio de Janeiro. One-year exposure to other injury or shocking events was nearly two-fold higher in Rio de Janeiro than in Sao Paulo (23.7% vs. 12.8%) – this difference was due to the proportion of participants who witnessed a shoot-out or were a victim of a stray bullet, which were nearly six times higher in Rio de Janeiro. Participants from Rio de Janeiro also reported witnessing more atrocities, slaughter or massacres, as well as witnessing attacks perpetrated by crime organizations.

The number of events reported by participants exposed to traumatic experiences varied from 1 to 19 (lifetime) and from 1 to 12 (one-year). Almost a half of respondents reporting exposure to traumatic events experienced four or more events in their lifespan. The proportion of those who reported four or more events was higher in Rio de Janeiro than São Paulo (54.9% vs. 47.2%, p<0.001). The mean number of events in the lifespan was higher in Rio de Janeiro (mean = 4.4; 95% CI: 4.2–4.5) than in Sao Paulo (mean = 3.9; 95% CI: 3.8–4.0). The mean number of events in the year prior to the interview was similar in the two cities (Sao Paulo = 1.5; 95% CI: 1.4–1.6; Rio de Janeiro = 1.6; 95% CI: 1.5–1.7).

Prevalence estimate of assaultive violence in the lifetime was higher among men than women (64.3% vs. 58%; p = 0.002); one-year prevalence estimates were similar in both genders (9.2% vs. 10.7%; p = 0.229). Men were more exposed to other injury or shocking events than women both in their lifetime (81.4% vs. 71.3%; p<0.001) and in the year prior to the interview (22.5% vs. 13.7%; p<0.001). Lifetime prevalence estimate of sudden death/life-threatening illness/injury of a close person was higher among females than males (50.5% vs. 44.3%; p = 0.002). One-year prevalence estimates were similar in both genders (5.5% vs. 5.3%; p = 0.808). Lifetime exposure to any traumatic event was higher among males than females in Sao Paulo (89.5% vs. 84.1%, p<0.001). The difference was not significant in Rio de Janeiro (91.4% vs. 87.8%, p = 0.058). One-year exposure was higher among men than women in Rio de Janeiro (42.3% vs. 31.6%, p<0.001), but not in Sao Paulo (24.4% vs. 21.4%, p = 0.145).

Additional information on the distribution of traumatic events across the main demographic characteristics of the sample is provided as Table S1.

### Prevalence Estimates of Psychiatric Disorders

Lifetime prevalence estimates of any psychiatric disorders were 44.0% in Sao Paulo and 42.1% in Rio de Janeiro (Table 4). One-year prevalence estimates of any psychiatric disorders were 32.5% in Sao Paulo and 31.2% in Rio de Janeiro. None of the prevalent estimates were statistically different between the two sites, with the exception of panic disorder (one-year), which was higher in Rio de Janeiro (1% vs. 0.1%, p<0.001). Lifetime prevalence estimates of any anxiety disorders were much higher among females than males in Sao Paulo (39.1% vs. 18.9%; p<0.001) and also in Rio de Janeiro (27.7% vs. 18%; p<0.001). One-year estimates of any anxiety disorders were also much higher for females than males (28.5% vs. 10.1% in Sao Paulo, and 23.3% vs. 11% in Rio de Janeiro). Lifetime prevalence estimate of major depressive disorder was nearly twice as high for females than males (Sao Paulo: 24.2% vs. 11.6%; p<0.001. Rio de Janeiro: 22.5% vs. 10.8%; p<0.001). One-year prevalence estimates of major depressive disorder were also much higher among females in Sao Paulo (11.7% vs. 3.4%; p<0.001) and Rio de Janeiro (8.8% vs. 2.4%; p<0.001). Lifetime prevalence estimates of alcohol dependence were higher for males than females in Sao Paulo (13.6% vs. 4.2%) and in Rio de Janeiro (14.9% vs. 5.2%). Lifetime prevalence estimates of post-traumatic stress disorder among females were 14.7% in Sao Paulo and 11.1% in Rio de Janeiro. One-year prevalence estimates of post-traumatic stress disorder in females were 7.8% in Sao Paulo and 4.7% in Rio de Janeiro.

**Table 4:** Weighted Prevalence of Psychiatric Disorders in Sao Paulo and Rio de Janeiro, Brazil, in 2007–2008 Stratified by Gender.

	Sao Paulo				Rio de Janeiro			
	Male	Female	TOTAL		Male	Female	TOTAL	
**Lifetime mental disorders**	**% (95% CI)**	**% (95% CI)**	**p**		**% (95% CI)**	**% (95% CI)**	**P**	
Alcohol hazardous use	13.6 (11.1–16.1)	4.2 (2.9–5.5)	<0.001	8.1 (6.8–9.5)	14.9 (11.4–18.3)	5.2 (3.4–7.0)	<0.001	9.4 (7.5–11.2)
Alcohol dependence	9.3 (7.1–11.5)	3.3 (2.2–4.4)	<0.001	5.8 (4.7–7.0)	8.8 (6.0–11.6)	4.3 (2.6–5.9)	<0.003	6.2 (4.7–7.7)
Major depressive disorder	11.6 (9.1–14.0)	24.2 (21.5–27.0)	<0.001	19.9 (17.0–20.8)	10.8 (8.8–15.0)	22.5 (21.4–28.5)	<0.001	17.4 (15.0–19.8)
Dysthymia	0.7 (0.1–1.2)	1.9 (1.0–2.7)	0.026	1.4 (0.8–1.9)	1.9 (0.6–3.2)	1.8 (0.7–3.0)	0.886	1.9 (1.0–2.7)
*Any Anxiety/phobic disorders*	18.9 (16.0–21.9)	39.1 (35.9–42.3)	<0.001	30.8 (28.5–33.1)	18.0 (14.4–21.6)	34.2 (30.2–38.1)	<0.001	27.7 (24.9–30.5)
Panic disorder	0.6 (0–1.3)	0.7 (0.3–1.3)	0.685	0.7 (0.3–1.1)	0.4 (0.0–0.9)	2.0 (0.8–3.4)	0.015	1.3 (0.6–2.1)
Specific phobia	9.6 (7.4–11.7)	22.0 (19.3–24.7)	<0.001	16.8 (14.9–18.6)	9.3 (6.6–12.0)	18.7 (15.5–22.0)	<0.001	14.6 (12.5–16.8)
Social phobia	3.9 (2.4–5.4)	7.0 (5.3–8.6)	0.008	5.7 (4.5–6.8)	2.6 (1.2–4.1)	5.1 (3.3–6.9)	0.038	4.0 (2.8–5.2)
Agoraphobia	1.6 (0.7–2.4)	5.9 (4.3–7.4)	<0.001	4.0 (3.1–5.0)	2.5 (1.1–3.9)	4.3 (2.7–5.9)	0.112	3.5 (2.4–4.6)
Generalized anxiety disorder	4.0 (2.5–5.5)	7.4 (5.6–9.1)	0.006	6.0 (4.7–7.1)	3.8 (2.0–5.5)	7.4 (5.2–9.7)	0.015	5.8 (4.3–7.3)
Obsessive-compulsive disorder	2.1 (1.1–3.2)	5.5 (4.1–7.0)	<0.001	4.1 (3.1–5.1)	2.5 (1.0–4.0)	4.5 (2.6–6.4)	0.107	3.6 (2.4–4.9)
Post-traumatic stress disorder	4.2 (2.7–5.6)	14.5 (12.2–16.9)	<0.001	10.2 (8.7–11.7)	5.5 (3.3–7.7)	11.1 (8.6–13.7)	0.002	8.7 (7.0–10.4)
Any disorder	37.5 (33.8–41.1)	48.7 (45.5–52.0)	<0.001	44.0 (41.6–46.4)	36.1 (31.6–40.7)	46.6 (42.4–50.8)	<0.001	42.1 (39.0–45.2)
**One-year mental disorders**								
Alcohol hazardous use	1.8 (0.9–2.6)	0.8 (0.2–1.3)	0.045	1.2 (0.7–1.6)	2.5 (1.1–3.8)	1.1 (0.3–1.9)	0.074	1.7 (0.9–2.4)
Alcohol dependence	3.1 (1.7–4.5)	1.5 (0.7–2.3)	0.030	2.2 (1.0–2.9)	2.0 (0.8–3.2)	0.3 (0.0–0.9)	0.028	1.0 (0.4–1.7)
Major depressive disorder	3.4 (2.0–4.7)	11.7 (9.7–13.8)	<0.001	8.2 (6.9–9.5)	2.4 (0.9–3.9)	8.8 (6.4–11.7)	<0.001	6.0 (4.5–7.5)
Dysthymia	0.4 (0.0–0.8)	1.1 (0.4–1.9)	0.130	0.8 (0.3–1.3)	1.1 (0.1–2.2)	0.5 (0.0–1.0)	0.227	0.8 (0.2–1.3)
*Any Anxiety/phobic disorders*	10.1 (7.9–12.2)	28.5 (25.6–31.5)	<0.001	20.8 (18.8–22.8)	11.0 (8.1–13.9)	23.6 (20.0–27.1)	<0.001	18.8 (16.3–21.2)
Panic disorder	0.1 (0.0–0.2)	0.2 (0.0–0.4)	0.211	0.1 (0.0–0.2)	0.4 (0.0–0.9)	1.5 (0.4–2.6)	0.069	1.0 (0.3–1.7)
Specific phobia	4.0 (2.7–5.3)	14.7 (12.3–17.0)	<0.001	10.2 (8.7–11.7)	5.4 (3.3–7.5)	12.9 (10.2–15.7)	<0.001	9.6 (7.8–11.5)
Social phobia	2.0 (0.9–3.1)	4.3 (3.0–5.5)	0.018	3.3 (2.4–4.2)	1.6 (0.4–2.8)	2.4 (1.2–3.7)	0.366	2.1 (1.2–2.9)
Agoraphobia	0.6 (0.1–1.1)	4.1 (2.8–5.5)	<0.001	2.7 (1.8–3.5)	1.8 (0.7–2.9)	2.3 (1.0–3.5)	0.568	2.1 (1.2–2.9)
Generalized anxiety disorder	1.8 (0.8–2.8)	4.8 (3.3–6.2)	0.002	3.5 (2.6–4.5)	1.3 (0.3–2.2)	3.0 (1.5–4.4)	0.055	2.2 (1.3–3.1)
Obsessive-compulsive disorder	1.5 (0.6–2.4)	4.6 (3.3–6.0)	,0.001	3.3 (2.5–4.2)	1.7 (0.5–2.9)	3.6 (2.0–5.3)	0.065	2.8 (1.7–3.9)
Post-traumatic stress disorder	1.2 (0.6–1.8)	7.8 (6.0–9.5)	<0.001	5.0 (3.9–6.1)	1.5 (0.4–2.6)	4.7 (3.0–6.4)	0.004	3.3 (2.2–4.4)
Any disorder	21.3 (18.2–24.3)	40.7 (37.5–43.9)	<0.001	32.5 (30.2–34.8)	22.3 (18.3–26.2)	38.1 (34.0–42.4)	<0.001	31.2 (28.3–34.1)

Abbreviations: CI, Confidence Interval; SP, Sao Paulo; RJ, Rio de Janeiro; p, Pearson Chi-Square Design-based (adjusted for design effect).

Additional information on the distribution of psychiatric diagnoses across the main demographic characteristics of the sample is provided as Tables S2 (lifetime) and S3 (one-year).

### Socio-demographic Correlates of Psychiatric Disorders

Table 5 shows the association between demographic characteristics and one-year psychiatric disorders, controlling for number of traumatic events reported by participants. As compared to those living in Sao Paulo, people living in Rio de Janeiro were more likely to have panic disorder (OR = 4.03; p = 0.014), and less likely to have alcohol dependence (OR = 0.53; p = 0.027), generalized anxiety disorder (OR = 0.53; p = 0.005), major depressive disorder (OR = 0.55; p<0.001) and post-traumatic stress disorder (OR = 0.62; p = 0.025). Women were less likely to have hazardous alcohol use (OR = 0.57; p = 0.043) and alcohol dependency (OR = 0.47; OR = 0.027) as compared to men. As compared to men, women were more likely to have specific phobia (OR = 3.22; p<0.001), social phobia (OR = 2.94; p<0.001), agoraphobia (OR = 3.15; p<0.001), generalized anxiety disorder (OR = 2.83; p<0.001), major depressive disorder (OR = 4.29; p<0.001) and post-traumatic stress disorder (OR = 4.58; p<0.001).

**Table 5:** Multivariate Logistic Regression of the Association Between One-Year DSM-IV PTSD and Non-Psychotic Psychiatric Disorders and Demographic Characteristics, controlled by trauma exposure, Sao Paulo and Rio de Janeiro, Brazil, 2007–2008.

	Alcohol hazardous use	Alcohol dependence	Panic disorder	Specific phobia	Social phobia	Agoraphobia	Obsessive-compulsive disorder	Generalized anxiety disorder	Major depressive disorder	Dysthymia	Post-traumatic stress disorder
	OR (95% CI)	OR (95% CI)	OR (95% CI)	OR (95% CI)	OR (95% CI)	OR (95% CI)	OR (95% CI)	OR (95% CI)	OR (95% CI)	OR (95% CI)	OR (95% CI)
**Setting**											
Sao Paulo	1	1	1	1	1	1	1	1	1	1	1
Rio de Janeiro	1.25 (0.66–2.38)	0.53 (0.30–0.93)	4.03 (1.33–12.2)	0.95 (0.73–1.24)	0.67 (0.41–1.08)	0.95 (0.52–1.72)	0.75 (0.47–1.22)	0.53 (0.34–0.82)	0.54 (0.38–0.78)	0.98 (0.42–2.27)	0.62 (0.41–0.94)
Trauma exposure*	1.15 (1.08–1.22)	1.34 (1.25–1.45)	1.32 (1.15–1.53)	1.13 (1.09–1.16)	1.25 (1.19–1.32)	1.27 (1.20–1.34)	1.23 (1.17–1.29)	1.19 (1.12–1.26)	1.20 (1.15–1.24)	1.28 (1.17–1.40)	1.30 (1.24–1.36)
**Gender**											
Male	1	1	1	1	1	1	1	1	1	1	1
Female	0.57 (0.33–0.98)	0.47 (0.29–0.76)	3.13 (0.88–11.1)	3.22 (2.45–4.21)	2.93 (1.73–4.98)	3.14 (1.77–5.58)	3.23 (2.15–4.85)	2.83 (1.82–4.41)	4.29 (3.05–6.02)	1.75 (0.70–4.36)	4.58 (2.83–7.42)
**Age**											
15–29	1	1	1	1	1	1	1	1	1	1	1
30–44	0.52 (0.28–0.97)	0.51 (0.27–0.99)	2.82 (0.68–11.7)	0.79 (0.60–1.05)	1.27 (0.75–2.13)	1.98 (1.07–3.67)	0.89 (0.52–1.51)	1.61 (0.87–2.98)	1.18 (0.85–1.63)	1.91 (0.46–7.90)	1.36 (0.89–2.06)
45–59	0.44 (0.19–1.02)	0.50 (0.22–1.13)	2.27 (0.32–16.0)	0.89 (0.65–1.21)	0.94 (0.50–1.77)	1.35 (0.70–2.63)	0.56 (0.33–0.98)	2.24 (1.23–4.07)	1.15 (0.79–1.65)	4.67 (1.08–20.3)	2.01 (1.25–3.23)
60–75	0.16 (0.03–0.83)	Dropped	2.21 (0.38–13.0)	0.59 (0.37–0.94)	0.27 (0.09–0.80)	0.77 (0.24–2.51)	0.18 (0.07–0.47)	1.75 (0.74–4.17)	0.56 (0.31–1.09)	2.68 (0.40–18.1)	0.73 (0.36–1.48)
Education (years)	0.89 (0.84–0.95)	0.89 (0.83–0.96)	0.88 (0.76–1.02)	0.96 (0.93–0.98)	0.94 (0.90–0.99)	0.91 (0.86–0.97)	0.94 (0.89–0.99)	1.01 (0.97–1.06)	0.98 (0.94–1.02)	0.93 (0.84–1.04)	0.92 (0.89–0.96)
**Marital status**											
Single	1	1	1	1	1	1	1	1	1	1	1
Married	0.66 (0.09–5.03)	Dropped	0.44 (0.56–3.44)	0.81 (0.46–1.43)	0.86 (0.29–2.55)	0.69 (0.19–2.42)	1.57 (0.65–3.76)	0.78 (0.36–1.70)	0.40 (0.43–3.77)	1.17 (0.25–5.37)	0.82 (0.38–1.79)
Divorced	1.22 (0.48–3.12)	1.36 (0.55–3.38)	0.38 (0.52–2.79)	1.22 (0.89–1.67)	1.04 (0.56–1.92)	0.89 (0.45–1.77)	1.77 (0.98–3.21)	1.06 (0.71–1.57)	1.27 (0.54–2.94)	0.75 (0.20–2.82)	0.82 (0.47–1.42)
Widowed	1.25 (0.68–2.29)	1.38 (0.79–2.42)	1.15 (0.35–3.77)	0.97 (0.75–1.27)	1.13 (0.73–1.76)	1.29 (0.74–2.24)	1.62 (1.05–2.50)	0.93 (0.71–1.21)	0.71 (0.42–1.20)	1.27 (0.45–3.53)	1.03 (0.70–1.50)
Employment	1.27 (0.71–2.27)	0.97 (0.55–1.69)	0.35 (0.14–0.89)	0.90 (0.70–1.17)	0.94 (0.62–1.41)	0.54 (0.34–0.87)	0.79 (0.54–1.16)	0.77 (0.58–1.02)	0.79 (0.47–1.34)	0.84 (0.35–2.02)	0.65 (0.47–0.89)
Migration	0.55 (0.28–1.10)	0.92 (0.47–1.81)	1.04 (0.43–2.50)	0.92 (0.73–1.16)	1.29 (0.84–2.00)	1.24 (0.76–2.06)	1.38 (0.93–2.04)	0.84 (0.63–1.14)	1.05 (0.97–1.14)	1.19 (0.51–2.79)	1.39 (0.97–1.99)

Abbreviations: OR, Odds Ratio; CI, Confidence Interval.

* Trauma exposure: number of different types of traumatic events.

As compared to those aged 15 to 29 years, participants aged 30 to 44 were more likely to have agoraphobia (OR = 1.98; p = 0.029), and less likely to have alcohol hazardous use (OR = 0.52; p = 0.041) and alcohol dependence (OR = 0.51; p = 0.046); those in the 45–59 age group were more likely to have generalized anxiety disorder (OR = 2.24; p = 0.009), dysthymia (OR = 4.61; p = 0.041) and post-traumatic stress disorder (OR = 2.01; p = 0.004), and less likely to have obsessive-compulsive disorder (OR = 0.57; p = 0.044); and participants aged 60 to 75 were less likely to have alcohol hazardous use (OR = 0.16; p = 0.029), specific phobia (OR = 0.59; p = 0.027), social phobia (OR = 0.027; p = 0.019) and obsessive-compulsive disorder (OR = 0.18; p = 0.001).

Participants who were currently employed were less likely to have panic disorder (OR = 0.35; p = 0.027), agoraphobia (OR = 0.54; p = 0.011) and post-traumatic stress disorder (OR = 0.65; p = 0.008) as compared to those who were unemployed.

There was a negative association between education and alcohol hazardous use (OR = 0.89; p = 0.001), alcohol dependence (OR = 0.089; p = 0.002), specific phobia (OR = 0.96; p = 0.001), social phobia (OR = 0.094; p = 0.035), agoraphobia (OR = 0.091; p = 0.003), obsessive-compulsive disorder (OR = 0.94; p = 0.029) and post-traumatic stress disorder (OR = 0.93; p<0.001), meaning that the higher the education, the lower the likelihood of having one of these diagnoses.

This logistic regression analysis also shows that there is a positive association between number of traumatic events reported by respondents and the prevalence estimates of all diagnoses, meaning that the higher the number of traumatic events experienced, the higher the likelihood of having alcohol hazardous use (OR = 1.15; p<0.001), alcohol dependence (OR = 1.34; p<0.001), panic disorder (OR = 1.32; p<0.001), specific phobia (OR = 1.13; p<0.001), social phobia (OR = 1.25; p<0.001), agoraphobia (OR = 1.27; p<0.001), generalized anxiety disorder (OR = 1.19; p<0.001), obsessive-compulsive disorder (OR = 1.23; p<0.001), major depressive disorder (OR = 1.2; p<0.001), dysthymia (OR = 1.28; p<0.001) and post-traumatic stress disorder (OR = 1.3; p<0.001).

### Association between Traumatic Events and Psychiatric Disorders

Table 6 shows the bivariate association of each type of traumatic events with psychiatric disorders. Assaultive violence and other injury or shocking events correlated with all psychiatric diagnoses, expect for panic disorder, whereas sudden death or life-threatening injury of a close person correlated with specific phobia (OR = 1.4; p<0.001), social phobia (OR = 2.35; p<0.001), obsessive-compulsive disorder (OR = 1.56; p = 0.016) and post-traumatic stress disorder (OR = 2.2; p<0.001).

**Table 6:** Bivariate Association Between One-Year DSM-IV Non-Psychotic Psychiatric Disorders and Types of Traumatic Events, Sao Paulo and Rio de Janeiro, Brazil, 2007–2008.

	Assaultive violence				Other injury				Sudden death			
	No	Yes	O. R.	P*	No	Yes	O. R.	P*	No	Yes	O. R.	P*
Alcohol hazardous use	18 (1.1%)	44 (2.1%)	1.94	0.017	9 (0.9%)	53 (2%)	2.29	0.019	37 (1.7%)	25 (1.6%)	0.94	0.810
Alcohol dependence	9 (0.6%)	57 (2.7%)	5.09	<0.001	10 (1.0%)	56 (2.1%)	2.18	0.002	33 (1.5%)	33 (2.1%)	1.4	0.174
Panic disorder	5 (0.3%)	10 (0.5%)	1.57	0.405	1 (0.1%)	14 (0.5%)	5.4	0.068	7 (0.3%)	8 (0.5%)	1.59	0.365
Specific phobia	142 (8.6%)	240 (11.5%)	1.37	0.005	83 (8%)	299 (11.1%)	1.43	0.005	189 (8.7%)	193 (12.4%)	1.48	<0.001
Social phobia	38 (2.3%)	73 (3.5%)	1.53	0.035	15 (1.5%)	96 (3.6%)	2.51	<0.001	42 (1.9%)	69 (4.4%)	2.35	<0.001
Agoraphobia	24 (1.5%)	63 (3%)	2.1	0.002	10 (1.0%)	77 (2.9%)	3.02	<0.001	42 (1.9%)	45 (2.9%)	1.51	0.058
Generalized anxiety disorder	30 (1.8%)	80 (3.8%)	2.14	<0.001	16 (1.5%)	94 (3.5%)	2.3	0.002	55 (2.5%)	55 (3.5%)	1.41	0.077
Obsessive-compulsive disorder	32 (2%)	88 (4.2%)	2.21	<0.001	12 (1.2%)	108 (4%)	3.56	<0.001	57 (2.6%)	63 (4%)	1.56	0.016
Major depressive disorder	92 (5.6%)	198 (9.5%)	1.76	<0.001	45 (4.3%)	247 (9.1%)	2.2	<0.001	147 (6.8%)	143 (9.2%)	1.39	0.007
Dysthymia	6 (0.4%)	21 (1%)	2.77	0.022	2 (0.2%)	25 (0.9%)	4.84	0.018	10 (0.5%)	17 (1.1%)	2.38	0.025
Post-traumatic stress disorder	39 (3.4%)	127 (6.1%)	1.83	<0.001	12 (2.2%)	154 (5.7%)	2.66	<0.001	53 (3.2%)	113 (7.2%)	2.39	<0.001

* P-value based on Pearson’s Chi-square test.

Table 7 shows the multivariate logistic regression models of the association between types of traumatic events and psychiatric diagnoses. When, controlled for demographic characteristics (Model 1), the bivariate associations found between assaultive violence and psychiatric disorders, and between other injuries or shocking events and psychiatric disorders remained statistically significant. The associations of sudden death or injury of a close person with major depressive disorder (p = 0.054) and with dysthymia (p = 0.056) turned into marginally significant.

**Table 7:** Association of types and number of traumatic events with one-year psychiatric disorders through multivariate logistic regression models.

	MODEL 1*		MODEL 2†	
ASSAULTIVE VIOLENCE				
	O. R. (95% CI)	p	O. R. (95% CI)	p
Alcohol hazardous use	2.29 (1.15–4.55)	0.019	2.02 (1.01–4.01)	0.046
Alcohol dependence	6.27 (3.07–12.8)	<0.001	5.66 (2.66–12.1)	<0.001
Specific phobia	1.53 (1.25–1.87)	<0.001	1.37 (1.11–1.69)	0.004
Social phobia	1.69 (1.11–2.55)	0.014	1.32 (0.87–1.99)	0.192
Agoraphobia	2.58 (1.49–4.48)	0.001	2.06 (1.2–3.53)	0.009
Generalized anxiety disorder	1.98 (1.22–3.19)	0.006	1.71 (1.45–2.78)	0.033
Obsessive-compulsive disorder	2.69 (1.8–4.03)	<0.001	2.1 (1.37–3.21)	0.001
Major depressive disorder	1.96 (1.46–2.62)	<0.001	1.66 (1.23–2.24)	0.001
Dysthymia	2.86 (1.03–7.93)	0.044	2.14 (0.82–5.59)	0.122
Post-traumatic stress disorder	2.14 (1.44–3.19)	<0.001	2.12 (1.41–3.17)	<0.001
**OTHER INJURIES OR SHOCKING EVENTS**				
	O. R. (95% CI)	p	O. R. (95% CI)	p
Alcohol hazardous use	2.36 (1.07–5.22)	0.034	1.97 (0.92–4.2)	0.079
Alcohol dependence	2.16 (1.09–4.3)	0.028	1.32 (0.63–2.74)	0.455
Specific phobia	1.6 (1.22–2.11)	0.001	1.38 (1.03–1.84)	0.033
Social phobia	2.83 (1.59–5.05)	0.001	2.2 (1.21–3.99)	0.010
Agoraphobia	3.66 (1.81–7.4)	<0.001	2.9 (1.42–5.9)	0.004
Generalized anxiety disorder	2.28 (1.27–4.08)	0.006	1.93 (1.07–3.49)	0.030
Obsessive-compulsive disorder	4.24 (2.32–7.74)	<0.001	3.3 (1.72–6.32)	<0.001
Major depressive disorder	2.45 (1.7–2.52)	<0.001	2.09 (1.43–3.05)	<0.001
Dysthymia	6.08 (1.16–22.2)	0.031	3.59 (0.9–14.3)	0.07
Post-traumatic stress disorder	3.15 (1.8–5.51)	<0.001	3.01 (1.73–5.25)	<0.001
**SUDDEN DEATH OR LIFE-THREATENING INJURY OF A CLOSE PERSON**				
	O. R. (95% CI)	p	O. R. (95% CI)	P
Specific phobia	1.43 (1.17–1.76)	0.001	1.29 (1.04–1.6)	0.020
Social phobia	2.35 (1.59–3.47)	<0.001	1.98 (1.33–2.96)	0.001
Obsessive-compulsive disorder	1.6 (1.09–2.35)	0.017	1.23 (0.84–1.8)	0.289
Major depressive disorder	1.29 (1.0–1.66)	0.054	1.07 (0.83–1.39)	0.577
Dysthymia	2.1 (0.98–4.87)	0.056	1.64 (0.75–3.6)	0.213
Post-traumatic stress disorder	2.2 (1.6–3.02)	<0.001	2.12 (1.41–3.17)	<0.001

* Model 1 = traumatic events controlling for demographic characteristics (city, gender, age, education, marital status, employment status, migration history).

† Model 2 = Model 1 plus other types of traumatic events.

Abbreviations: O. R. = Odds Ration; p = p value; CI = confidence interval.

When the three types of traumatic events were included in the same logistic regression model (Model 2), together with demographic characteristics, assaultive violence remained associated with alcohol hazardous use (OR = 2.02; p = 0.046), alcohol dependence (OR = 5.66; p<0.001), specific phobia (OR = 0.37; p = 0.004), agoraphobia (OR = 2.06; p = 0.009), generalized anxiety disorder (OR = 1.71; p = 0.033), obsessive-compulsive disorder (OR = 2.1; p = 0.001), major depressive disorder (OR = 1.66; p = 0.001), and post-traumatic stress disorder (OR = 2.12; p<0.001). Other injury or shocking events remained associated with specific phobia (OR = 1.38; p = 0.033), social phobia (OR = 2.2; p = 0.010), agoraphobia (OR = 2.9; p = 0.004), generalized anxiety disorder (OR = 1.93; p = 0.030), obsessive-compulsive disorder (OR = 3.3; p<0.001), major depressive disorder (OR = 2.09; p<0.001), and post-traumatic stress disorder (OR = 3.01; p<0.001). Sudden death/life-threatening illness or injury of a close person remained associated with specific phobia (OR = 1.29; p = 0.020), social phobia (OR = 1.98; p = 0.001), and post-traumatic stress disorder (OR = 2.12; p<0.001).

## Discussion

Exposure to traumatic events in the two surveyed cities is highly prevalent: nearly 90% of people living in Sao Paulo and Rio de Janeiro faced at least one lifetime traumatic experience. Moreover, one-third of the population in Rio de Janeiro and one-fifth in Sao Paulo were exposed to at least one traumatic event in the year prior to the interview. If we take into consideration only those events regarded as direct exposure to violence (assaultive violence), 59.4% of residents in Sao Paulo and 63.4% in Rio de Janeiro reported a lifetime exposure. The one-year prevalence estimates of such events were 9.5% in Sao Paulo and 11.4% in Rio de Janeiro. Although the overall lifetime prevalence estimates of traumatic events were similar in both cities, the occurrence of multiple episodes was more frequent in Rio de Janeiro.

Compared to other Latin American countries whose levels of development are similar to Brazil’s, exposure to traumatic events was higher in the current study than in Mexico [17], [18], [29] and Chile [19], [30]. These differences may be due to the fact that the current study used a much more comprehensive list of traumatic events. When it was possible to compare events that were assessed in Brazil, Mexico and Chile, Brazilians had slightly higher rates of exposure to community violence, i.e. violence occurring outside the family environment, whereas violence usually perpetrated by close persons, such as parental, intimate-partner and sexual violence was more prevalent in Mexico and Chile.

Our results also show that psychiatric disorders are common in Sao Paulo and Rio de Janeiro. More than 40% of participants had at least one lifetime psychiatric disorder, and nearly a third of them had at least a one-year diagnosis. Our one-year prevalence estimate was slightly higher than the 29.6% reported by Andrade et al (2012) in a study carried out in Sao Paulo Metropolitan Area [14]. It is noteworthy that, except for post-traumatic stress disorder, one-year prevalence estimates of all diagnostic categories in our Sao Paulo sample were similar to those found in Sao Paulo Metropolitan Area. Prevalence estimates of alcohol related disorders and phobic-anxiety disorders in Rio de Janeiro were also similar to those reported by Andrade et al, whereas major depressive disorder was less prevalent in Rio de Janeiro (9.4% vs. 6%). One-year prevalence estimates of post-traumatic stress disorder were significantly higher in our study (5% in Sao Paulo and 3.3% in Rio de Janeiro) than in the Sao Paulo Metropolitan Area (1.6%). These differences may be due to methodological issues, as Andrade and colleagues assessed post-traumatic stress symptoms only among those who screened positive for any other disorders and a random sample of those who screened negative [14], whereas we applied the post-traumatic stress disorder section to all respondents. Moreover, by using a more comprehensive list of traumatic events, our study may have assessed symptoms for a higher proportion of participants who endorsed at least one traumatic event.

Prevalence estimates of psychiatric disorders in our study were also higher in Sao Paulo and Rio de Janeiro than in Mexico and Chile, where the prevalence estimates of alcohol abuse/dependence, anxiety disorders and major depressive disorder were found to be 11%, 14% and 7.2% (Mexico) [29] and 10%, 16.2% and 9.2% (Chile) [30]. When compared to high-income countries [31], [32], our study found lifetime prevalence estimates of alcohol abuse/dependence (13.9% in Sao Paulo; 15.6% in Rio de Janeiro) to be lower than in the USA (18.6%) and almost three times as high as in Europe (5.2%). Prevalence estimates of anxiety disorders in our study, including post-traumatic stress disorder (30.8% in Sao Paulo; 27.1% in Rio de Janeiro) were similar to the USA’s estimates (28.8%), and more than twice as high as Europe’s figures (13.6%). Major depressive disorder was more prevalent in Sao Paulo (19.9%) and Rio de Janeiro (17.4%) than in the USA (16.6%) and Europe (13.6%).

### Demographic Correlates of Psychiatric Disorders

As it has been consistently reported in other epidemiological studies [14], [33], [34], [35], our results show that women were more likely to have phobic-anxiety disorders, obsessive compulsive disorder, major depressive disorder and post-traumatic stress disorder, and men were more likely to have alcohol related disorders, which confirms that internalizing disorders are more frequent among women, whereas externalizing disorders are more common among men [36]. We also found that older age was associated with a lower likelihood of alcohol-related disorders, phobic disorders, obsessive compulsive disorder and major depressive disorder, and that lower education correlated with higher prevalence estimates of alcohol-related disorders, specific phobia, agoraphobia and post-traumatic stress disorder. Other studies have also shown that prevalence estimates of most psychiatric disorders tend to decrease as age and education increase [14], [34]. Being divorced correlated with specific phobia and being unemployed correlated with agoraphobia and post-traumatic stress disorder.

Participants living in Rio de Janeiro had a higher likelihood of panic disorder, whereas living in Sao Paulo correlated with alcohol dependence, generalized anxiety disorder and major depressive disorder and post-traumatic stress disorder. These results may suggest that characteristics of the two cities may be mediating the relationship between traumatic events and mental disorders. One hypothesis is that higher exposure to violence in Rio de Janeiro may increase fear, whereas the city’s characteristics provides its inhabitants with a healthier life-style, which may improve quality of living and reduce levels of distress [37]. It has been proposed that panic disorder correlates with an underlying fear-factor, whereas generalized anxiety disorder, major depression and post-traumatic stress disorder share an underlying distress factor [38], [39].Further studies exploring the role of differences in standards of living and environmental characteristics of large urban centers would allow for the identification of modifiable risk and/or protective factors, leading to the implementation of large-scale preventive interventions, such as the promotion of a healthier life-style and changes in the cities’ infrastructure which might provide their inhabitants with a better quality of life.

### Association of Traumatic Events and Psychiatric Disorders

Results from our logistic regression models show that assaultive violence and other injury or shocking events correlated with all psychiatric disorders, expect for panic disorder, and that sudden death/life-threatening injury of a close person correlated with five out of the eleven diagnoses. When the analysis took into consideration the overlap between types of events by including the three classes of traumatic events in the same model, assaultive violence remained correlated with eight psychiatric disorders, other injury or shocking events with seven, and sudden death/life-threatening injury of a close person with three.

Results from the logistic regression models show that, as far as different types of traumatic experiences are concerned, assaultive violence may increase the vulnerability of developing alcohol-related disorders, phobic and anxiety disorders, including post-traumatic stress disorder, and major depressive disorder. Other injury or shocking events may increase the likelihood of developing phobic and anxiety disorder and major depressive disorder. Sudden-death/life-threatening injury or illness of a close person increased the likelihood of developing specific and social phobia, as well as post-traumatic stress disorder. These results suggest that interpersonal violence may affect mental health status more than other types of events, as it has been previously reported in other low and middle-income countries [18], [19], [40].

Our results also show a dose-response relationship between number of traumatic events and all eleven psychiatric disorders. This relationship confirms previous evidence that traumatic experiences may have a cumulative negative effect on mental health [15], [17], [20], [41], [42], giving support to the sensitization theory, according to which previously exposed people would show a greater responsiveness to subsequent stressors [43]. There are longitudinal data rendering support to this hypothesis such as a study conducted with adolescents exposed to multiple traumatic events showing that they were more likely to develop post-traumatic stress disorder or depression than those exposed to a single event [42]. Another longitudinal study found that a subsequent trauma increased the risk of post-traumatic stress disorder but only for those who had already developed post-traumatic stress disorder from a previous exposure [44]. It is also not possible to exclude a recall bias role since people with previous psychiatric disorders tend to evoke distressful experiences more frequently than healthy individuals for a comparable event [45]. Moreover, personal vulnerabilities, such as neuroticism and a past history of treatment, may be a stronger predictor than severity of trauma to develop an individual psychiatric response [46].

### Limitations

Due to the cross-sectional design of the current study, reverse causality may not be discarded when considering the association between traumatic events and mental disorders. Another limitation of this study is that the outcomes were based on the CIDI 2.1, which is a lay-administered questionnaire [35], [47]. Even though the questionnaire has proven to have satisfactory sensitivity and validity both in Brazil and abroad [27], [31], it can lead to a certain degree of misclassification due to both its nature and methodological shortcomings, such as recall bias. Additionally, this study was carried out in the two largest urban centers in Brazil. Due to the country’s heterogeneity, the results presented here may not apply to small and medium cities, which may have different patterns of social distribution and living conditions. This fact may partially explain the higher rates of psychiatric disorders found in this study as compared to surveys that included nationally representative samples, since it has been shown that people living in urban centers are at a higher risk of developing mental disorders as compared to those living in rural areas [32]. On the other hand, many metropolitan areas in Brazil and other LMIC share similar features, such as rapid urbanization, social inequality and ongoing epidemic levels of violence.

### Conclusion

Our findings show that psychiatric disorders and traumatic events, especially violence, are extremely common in Sao Paulo and Rio de Janeiro, supporting the idea that neuropsychiatric disorders and external causes have become a major public health priority, as they are amongst the leading causes of burden of disease in low and middle-income countries. [1], [4], [5].

By highlighting the differences between the two cities regarding their patterns of violence and psychiatric morbidity, our study suggests that the identification of environmental factors that may buffer the negative impacts of violence and other urban stressors might guide the implementation of interventions meant to improve quality of life in LMIC urban centers and, as a result, of the health profile of the populations living in large cities.

## Supporting Information

Table S1 **Weighted prevalence estimates of exposure to traumatic events in Sao Paulo and Rio de Janeiro, Brazil, stratified by demographics.**
(DOCX)

Table S2 **Weighted prevalence estimates of lifetime psychiatric disorders in Sao Paulo and Rio de Janeiro, Brazil, stratified by demographics.**
(DOCX)

Table S3 **Weighted prevalence estimates of one-year psychiatric disorders in Sao Paulo and Rio de Janeiro, Brazil, stratified by demographics.**
(DOCX)
