# Supplementary material for: The Impact of Epidemic Violence on the Prevalence of Psychiatric Disorders in Sao Paulo and Rio de Janeiro, Brazil
Source: PLoS One. 2013 May 8;8(5):e63545. doi: 10.1371/journal.pone.0063545 (PMC3648507; doi:10.1371/journal.pone.0063545)
Supplement: Table S1 — Weighted prevalence estimates of exposure to traumatic events in Sao Paulo and Rio de Janeiro, Brazil, stratified by demographics. (DOCX) [file pone.0063545.s001.docx]

Supplemental table 1: weighted exposure to traumatic events in Sao Paulo and Rio de Janeiro, Brazil in 2007-2008, by type of event, stratified by demographics

|  | **Assaultive violence** | | **Other injury or shocking events** | | **Sudden death/injury of a close person** | | **Any traumatic event** | |
| --- | --- | --- | --- | --- | --- | --- | --- | --- |
|  | **Lifetime** | **One-year** | **Lifetime** | **One-year** | **Lifetime** | **One-year** | **Lifetime** | **One-year** |
| **Gender** |  |  |  |  |  |  |  |  |
| Male | 64.3%* | 9.2% | 81.4%* | 22.5%* | 44.3%* | 5.3% | 90.1%* | 29.7%* |
| Female | 58.0% | 10.7% | 71.3% | 13.7% | 50.5% | 5.5% | 85.2% | 24.3% |
| **Age (years)** |  |  |  |  |  |  |  |  |
| 15-29 | 53.1%* | 13.9%* | 72.9%* | 22.4%* | 41.6%* | 6.6%* | 84.5%* | 33.4% |
| 30-44 | 65% | 8.8% | 78% | 17.5% | 49.7% | 6.3% | 88.9% | 26% |
| 45-59 | 65.2% | 8.1% | 80.5% | 15.9% | 50.8% | 4.4% | 90.5% | 24.2% |
| 60-74 | 59% | 8% | 66.7% | 9% | 52% | 2.9% | 83.6% | 17.4% |
| **Marital status** |  |  |  |  |  |  |  |  |
| Single | 54.2%* | 11.7% | 72.8%* | 20%* | 40.7%* | 6.6% | 82.9%* | 30%* |
| Married/cohabiting | 62.1% | 9,5% | 76.3% | 16.5% | 48.7% | 4.9% | 88.3% | 25.3% |
| Separated/divorced | 71.8% | 9.7% | 82.4% | 18.4% | 55.2% | 4.3% | 93% | 26.8% |
| Widowed | 60.9% | 7.5% | 71% | 11.1% | 63.7% | 6.6% | 89.3% | 21.6% |
| **Education (years of school)** |  |  |  |  |  |  |  |  |
| 0-4 | 48.8% | 7.3% | 64.4%* | 10.9%* | 44.8% | 4.3% | 79.3%* | 17.2%* |
| 5-8 | 55.9% | 9.5% | 75.4% | 16% | 46.5% | 6.2% | 86.1% | 26.2% |
| 9-12 | 63.4% | 11% | 77.3% | 22.2% | 48.2% | 6.2% | 89.3% | 31.4% |
| 13 or more | 74.1% | 11.5% | 84.3% | 15.5% | 52.3% | 3.8% | 93.1% | 26.1% |
| **Occupational status** |  |  |  |  |  |  |  |  |
| Unemployed | 55.1%* | 10.8% | 72.2%* | 15.3%* | 48.9% | 5.2% | 85.1%* | 25.7% |
| Currently employed | 64.5% | 9.5% | 77.9% | 18.9% | 47.1% | 5.6% | 88.7% | 27.2% |
| **Migration history** |  |  |  |  |  |  |  |  |
| No | 61.7% | 10.5% | 79.8%* | 20.5%* | 47.5% | 5.4% | 89.3%* | 29.5%* |
| Yes | 59.4% | 9.5% | 70.4% | 13.6% | 48.3% | 5.5% | 84.8% | 23% |
