# Supplementary material for: The Impact of Epidemic Violence on the Prevalence of Psychiatric Disorders in Sao Paulo and Rio de Janeiro, Brazil
Source: PLoS One. 2013 May 8;8(5):e63545. doi: 10.1371/journal.pone.0063545 (PMC3648507; doi:10.1371/journal.pone.0063545)
Supplement: Table S2 — Weighted prevalence estimates of lifetime psychiatric disorders in Sao Paulo and Rio de Janeiro, Brazil, stratified by demographics. (DOCX) [file pone.0063545.s002.docx]

Supplemental table 2: weighted prevalence of lifetime psychiatric disorders in Sao Paulo and Rio de Janeiro, Brazil in 2007-2008, by type of event, stratified by demographics

|  | **Alcohol hazardous use** | **Alcohol dependence** | **Panic disorder** | **Specific phobia** | **Social phobia** | **Agoraphobia** | **Obsessive-compulsive disorder** | **Generalized anxiety disorder** | **Major depressive disorder** | **Dysthymia** | **Post-traumatic stress disorder** |
| --- | --- | --- | --- | --- | --- | --- | --- | --- | --- | --- | --- |
| **Gender** |  |  |  |  |  |  |  |  |  |  |  |
| Male | 14%* | 9.2%* | 0.6% | 9.5%* | 3.5%* | 1.8%* | 2.3%* | 3.9%* | 11.4%* | 1% | 4.6%* |
| Female | 4.5% | 3.6% | 1.2% | 21.1% | 6.4% | 5.4% | 5.2% | 7.4% | 23.7% | 1.9% | 13.6% |
| **Age (years)** |  |  |  |  |  |  |  |  |  |  |  |
| 15-29 | 9.6%* | 5.9% | 0.7% | 17%* | 4.6%* | 2.9% | 3.8%* | 3.1%* | 16.3%* | 0.9%* | 7.3%* |
| 30-44 | 9.6% | 7.3% | 1.1% | 15% | 7.2% | 4.5% | 5.9% | 7.2% | 23.1% | 1.2% | 9.8% |
| 45-59 | 8.1% | 5.7% | 0.9% | 19.9% | 5.4% | 5% | 3.5% | 8.3% | 20.2% | 3% | 13.8% |
| 60-74 | 4.4% | 3.2% | 0.9% | 10.2% | 1.5% | 2.5% | 1% | 4.8% | 9.5% | 1% | 7.7% |
| **Marital status** |  |  |  |  |  |  |  |  |  |  |  |
| Single | 7.4% | 5.9% | 0.9% | 14.9% | 4.7% | 3.4% | 4.2% | 4.8% | 15.1%* | 0.8%* | 7.5%* |
| Married/cohabiting | 9.2% | 5.7% | 1% | 15.8% | 5.7% | 4.2% | 3.6% | 6.3% | 19.1% | 1.5% | 9.9% |
| Separated/divorced | 10.1% | 8.9% | 0.7% | 22.3% | 4.9% | 4.9% | 6.5% | 7.9% | 27.9% | 2.8% | 14.9% |
| Widowed | 4.8% | 3.5% | 0.4% | 16.3% | 3.4% | 1.9% | 1.9% | 5.1% | 14.3% | 3.4% | 11.6% |
| **Education (years of school)** |  |  |  |  |  |  |  |  |  |  |  |
| 0-4 | 6.9%* | 6.9% | 0.9%* | 18.2%* | 4.3% | 4.4%* | 3.8%* | 4.2% | 14.7% | 2.2% | 11%* |
| 5-8 | 10.1% | 6.1% | 0.5% | 18.4% | 7.2% | 5.7% | 3.3% | 5.6% | 19.5% | 1.2% | 11.7% |
| 9-12 | 8.1% | 6.2% | 0.5% | 16.4% | 4.8% | 2.9% | 5.2% | 6.6% | 18.7% | 1.8% | 9.5% |
| 13 or more | 8.7% | 4.2% | 2.4% | 10.4% | 4.2% | 2.9% | 2.3% | 6.8% | 20.8% | 0.6% | 6.4% |
| **Occupational status** |  |  |  |  |  |  |  |  |  |  |  |
| Unemployed | 5.8%* | 4.6%* | 1% | 18.5%* | 5.6% | 5.6%* | 4.5% | 5.3% | 18.4% | 1.9% | 11% |
| Currently employed | 10.4% | 6.8% | 0.8% | 14.5% | 4.9% | 2.7% | 3.7% | 6.4% | 18.6% | 1.3% | 8.9% |
| **Migration history** |  |  |  |  |  |  |  |  |  |  |  |
| No | 9.4% | 6.3% | 1% | 15.8% | 4.5% | 3.3% | 3.5% | 5.7% | 19.3% | 1.3% | 8.8% |
| Yes | 7.4% | 5.4% | 0.8% | 16.6% | 6% | 4.6% | 4.6% | 6.1% | 17.5% | 1.8% | 10.9% |
