# Supplementary material for: The Impact of Epidemic Violence on the Prevalence of Psychiatric Disorders in Sao Paulo and Rio de Janeiro, Brazil
Source: PLoS One. 2013 May 8;8(5):e63545. doi: 10.1371/journal.pone.0063545 (PMC3648507; doi:10.1371/journal.pone.0063545)
Supplement: Table S3 — Weighted prevalence estimates of one-year psychiatric disorders in Sao Paulo and Rio de Janeiro, Brazil, stratified by demographics. (DOCX) [file pone.0063545.s003.docx]

Supplemental table 3: weighted prevalence of one-year psychiatric disorders in Sao Paulo and Rio de Janeiro, Brazil in 2007-2008, by type of event, stratified by demographics

|  | **Alcohol hazardous use** | **Alcohol dependence** | **Panic disorder** | **Specific phobia** | **Social phobia** | **Agoraphobia** | **Obsessive-compulsive disorder** | **Generalized anxiety disorder** | **Major depressive disorder** | **Dysthymia** | **Post-traumatic stress disorder** |
| --- | --- | --- | --- | --- | --- | --- | --- | --- | --- | --- | --- |
| **Gender** |  |  |  |  |  |  |  |  |  |  |  |
| Male | 2%* | 2.8%* | 0.2%* | 4.4%* | 1.9%* | 1%* | 1.6%* | 1.7%* | 3.1%* | 0.6% | 1.3%* |
| Female | 0.8% | 1.2% | 0.6% | 14.2% | 3.8% | 3.6% | 4.4% | 4.3% | 10.9% | 1% | 6.9% |
| **Age (years)** |  |  |  |  |  |  |  |  |  |  |  |
| 15-29 | 2.5%* | 2.8%* | 0.001% | 11.1% | 2.4%* | 2% | 3.3%* | 1.8%* | 6.2%* | 0.5% | 3.1%* |
| 30-44 | 1% | 2.3% | 0.6% | 9.4% | 4.1% | 3.2% | 4.3% | 3.5% | 9.6% | 0.5% | 4.6% |
| 45-59 | 0.9% | 1.1% | 0.4% | 11.2% | 3.4% | 2.5% | 3% | 4.7% | 8.7% | 1.7% | 7.3% |
| 60-74 | 0.2% | 0% | 0.6% | 6.9% | 0.9% | 1.8% | 0.7% | 2.6% | 4.2% | 0.7% | 2.3% |
| **Marital status** |  |  |  |  |  |  |  |  |  |  |  |
| Single | 2.2%* | 2.5% | 0.2% | 9.4% | 2.7% | 2.4% | 3.5% | 2.7% | 6.2%* | 0.6% | 3.9% |
| Married/cohabiting | 1% | 1.7% | 0.5% | 9.6% | 3.2% | 2.7% | 2.8% | 3.5% | 7.7% | 0.8% | 4.7% |
| Separated/divorced | 1% | 2.2% | 0.3% | 13.8% | 3.5% | 2% | 5.3% | 3.8% | 11.8% | 0.7% | 5.8% |
| Widowed | 0.2% | 0% | 0.4% | 10.7% | 1.5% | 1.6% | 1.9% | 1.2% | 6.9% | 2.2% | 4.4% |
| **Education (years of school)** |  |  |  |  |  |  |  |  |  |  |  |
| 0-4 | 0.8% | 1.4%* | 0.7% | 10% | 2.1% | 2.2% | 3.5%* | 2.6% | 7.3% | 1.2% | 5.4% |
| 5-8 | 1.9% | 2.6% | 0.2% | 10.5% | 4.5% | 4% | 2.8% | 2.5% | 7.1% | 0.6% | 5% |
| 9-12 | 1.4% | 2.2% | 0.2% | 11.1% | 2.6% | 2.3% | 4.1% | 3.6% | 8.2% | 0.9% | 4.6% |
| 13 or more | 0.8% | 0.4% | 0.7% | 7.1% | 2.4% | 1.3% | 1.3% | 3.6% | 7.4% | 0.4% | 2.7% |
| **Occupational status** |  |  |  |  |  |  |  |  |  |  |  |
| Unemployed | 1.1% | 1.6% | 0.6% | 11.6%* | 2.9% | 3.9%* | 3.6% | 2.3% | 8.9%* | 1% | 6%* |
| Currently employed | 1.5% | 2% | 0.2% | 9% | 3% | 1.5% | 2.9% | 3.7% | 6.7% | 0.7% | 3.5% |
| **Migration history** |  |  |  |  |  |  |  |  |  |  |  |
| No | 1.8%* | 2.3% | 0.4% | 10.3% | 2.4% | 1.9%* | 2.8% | 3.1% | 8.1% | 0.6% | 3.6%* |
| Yes | 0.7% | 1.3% | 0.4% | 9.7% | 3.7% | 3.3% | 3.7% | 3.2% | 7% | 1.1% | 5.6% |
